# Case management-based post-stroke care for patients with acute stroke and TIA (SOS-Care): a prospective cohort study

**DOI:** 10.1007/s00415-024-12387-0

**Published:** 2024-06-14

**Authors:** Kristian Barlinn, Simon Winzer, Uwe Helbig, Falko Tesch, Lars-Peder Pallesen, Heike Trost, Nastasja Pfaff, Sandy Klewin, Daniela Schoene, Ulf Bodechtel, Jens Schwarze, Volker Puetz, Timo Siepmann, Bernhard Rosengarten, Heinz Reichmann, Jochen Schmitt, Jessica Barlinn

**Affiliations:** 1 Department of Neurology, Faculty of Medicine and University Hospital Carl Gustav Carus, Technische Universität Dresden, Fetscherstrasse 74, 01307 Dresden, Germany; 2 Center for Evidence-Based Healthcare, Faculty of Medicine and University Hospital Carl Gustav Carus, Technische Universität Dresden, Dresden, Germany; 3 Center for Intensive Care Rehabilitation, Klinik Bavaria, Kreischa, Germany; 4 Department of Neurology, Klinikum Chemnitz gGmbH, Chemnitz, Germany

**Keywords:** Post-stroke care, Secondary stroke prevention, Stroke recurrence, Vascular mortality

## Abstract

**Background:**

The high incidence of stroke recurrence necessitates effective post-stroke care. This study investigates the effectiveness of a case management-based post-stroke care program in patients with acute stroke and TIA.

**Methods:**

In this prospective cohort study, patients with TIA, ischemic stroke or intracerebral hemorrhage were enrolled into a 12-month case management-based program (SOS-Care) along with conventional care. Control patients received only conventional care. The program included home and phone consultations by case managers, focusing on education, medical and social needs and guideline-based secondary prevention. The primary outcome was the composite of stroke recurrence and vascular death after 12 months. Secondary outcomes included vascular risk factor control at 12 months.

**Results:**

From 11/2011 to 12/2020, 1109 patients (17.9% TIA, 77.5% ischemic stroke, 4.6% intracerebral hemorrhage) were enrolled. After 85 (7.7%) dropouts, 925 SOS-Care patients remained for comparative analysis with 99 controls. Baseline characteristics were similar, except for fewer males and less frequent history of dyslipidemia in post-stroke care. At 12 months, post-stroke care was associated with a reduction in the composite endpoint compared to controls (4.9 vs. 14.1%; HR 0.30, 95% CI 0.16–0.56, *p* < 0.001), with consistent results in ischemic stroke patients alone (HR 0.32, 95% CI 0.17–0.61, *p* < 0.001). Post-stroke care more frequently achieved treatment goals for hypertension, dyslipidemia, diabetes, BMI and adherence to secondary prevention medication (*p* < 0.05).

**Conclusions:**

Case management-based post-stroke care may effectively mitigate the risk of vascular events in unselected stroke patients. These findings could guide future randomized trials investigating the efficacy of case management-based models in post-stroke care.

## Introduction

Although major advances in stroke-reperfusion strategies have been achieved in the last few years, the health-related and socio-economic burden of the disease is still on the rise [1]. Recurrent strokes, occurring in about 10% of stroke survivors within the first year, are often linked to inadequate risk factor control and largely impact individual disability and long-term survival [2–4]. Therefore, increasing attention is led toward post-stroke control of modifiable vascular risk factors that together account for the vast majority of all strokes [5, 6]. However, nearly half of stroke survivors discharged from hospital or rehabilitation do not have appropriate access to specialized post-stroke services [7]. This deficiency is largely attributed to the absence of specialized stroke services in the outpatient real-world stroke-prevention setting [3, 8].

Inspired by successful patient-support programs for chronic diseases like coronary heart disease, several secondary stroke-prevention programs have recently been developed [7–13]. However, the INSPiRE-TMS trial, involving eight outpatient visits over two years for transient ischemic attack (TIA) and minor stroke patients, failed to demonstrate a reduction in recurrent stroke or vascular death, despite achieving secondary prevention targets [14]. In contrast, the STROKE-CARD trial, which provided a single outpatient visit and a web-based portal for stroke education and risk factor monitoring, showed lower stroke recurrence and vascular death rates in the intervention group, without a direct effect on risk factor control [15]. The SANO trial, with a focus on five outpatient visits, also failed to significantly reduce vascular events, despite effectively controlling certain risk factors [16].

Unlike these secondary stroke-prevention programs that largely focused on outpatient visits, a case management-based approach may provide more personalized and patient-centered care [10]. In a prior pilot case–control study, such post-stroke care model was associated with both vascular risk factor control and reduced risk of stroke recurrence [11]. The aim of the present study was to evaluate the effectiveness of case management-based post-stroke care in a prospective cohort of TIA and stroke patients.

## Methods

### Study design

This prospective cohort study included consecutive adult patients with acute cerebrovascular events admitted to two tertiary stroke centers in Saxony, Germany. The study population was non-randomly divided into two groups: (1) patients who received 12-month case management-based post-stroke care plus conventional care; and (2) those who received conventional care alone.

### Case management-based post-stroke care

The Stroke East Saxony post-stroke care (SOS-Care) program was initiated at the University Hospital Dresden in 12/2011 to provide comprehensive care to stroke patients discharged from hospital or rehabilitation to their homes [10, 11]. Eligible patients included those hospitalized for TIA, acute ischemic stroke or intracerebral hemorrhage. Patients with functional dependence pre-stroke (defined as degree of care ≥ 2 in the German healthcare system) [17], severe cognitive impairment from pre-known dementia and those receiving palliative care were deemed ineligible for the program. Initially, only patients with statutory health insurance with AOK were included. As one of the largest health insurers in Germany, the AOK played a crucial role as co-initiator and funder of the program [18]. In 07/2015, SOS-Care expanded to include the tertiary-care Municipal Hospital Chemnitz, which allowed enrollment of patients irrespective of their health insurance status as funding was provided entirely by the hospital. Originally facilitated by a single case manager, the program has since evolved to include four certified stroke-case managers.

Within the first three days of hospitalization, eligible patients received a standardized educational discussion from a case manager covering stroke, its association with vascular risk factors, recurrence risks, importance of secondary prevention and lifestyle changes including nicotine abstinence, weight management, healthy diet and physical activity. Patient proxies were included if needed. After consent for the post-stroke program, a case manager-led home visit was scheduled within one week of hospital or rehabilitation discharge to consolidate stroke education. This visit also included reassessment of the patients´ vascular risk profile, considering medical history, recent hospital and rehabilitation data and current body weight and blood pressure measurements. Follow-up phone consultations were conducted at 3, 6 and 9 months post-stroke to maintain education, monitor vascular risk factors and ensure adherence to secondary prevention medication including antiplatelets and oral anticoagulants. Patients´ self-measured weight and blood pressure were recorded during each phone-based visit. HbA1c and LDL cholesterol (LDL-C) levels were collected from primary care physicians at 6 and 12 months. If the patients´ treatment targets deviated from current European Stroke Organization guidelines, adjustments to medications were made in collaboration with the program’s stroke experts and primary care physicians [19]. In addition, the case managers coordinated necessary ambulatory care for patients´ medical and social health needs. Besides scheduled interactions, the case managers remained available for additional assistance via phone and email during work hours.

At 12 months, a final home visit was conducted to measure weight and blood pressure, assess adherence to secondary prevention medications and lifestyle changes, and obtain the modified Rankin scale (mRS) score. Due to the COVID-19 pandemic, final visits were conducted via phone since 03/2020 and self-measured weight and blood pressure values were provided by the patients. In addition, patients were sent surveys including the EuroQol 5D-3L (EQ-5D-3L) and Patient Health Questionnaire-9 (PHQ-9) via mail [20, 21]. If there was no response within 2 weeks, patients and their proxies were reminded by phone.

### Control patients

Consecutive patients who met the eligibility criteria for SOS-Care but were not insured by AOK qualified as controls and were prospectively recruited at the Dresden study site over a two-year period. To avoid information bias, study personnel other than case managers invited them to participate in the study. At 12 months, control patients were visited at home by a case manager or attended an outpatient visit to measure body weight and blood pressure, assess adherence to secondary prevention medication and lifestyle changes as well as to obtain mRS score. Laboratory data for HbA1c and LDL-C were provided by primary care physicians. EQ-5D-3L and PHQ-9 questionnaires were also mailed to controls at 12 months.

Both control and SOS-Care patients received conventional care including standard discharge discussions on vascular risk factor management, medications and lifestyle changes from treating physicians. There were no differences in acute or post-stroke care based on insurance status between the two groups.

### Study outcomes

The primary outcome was a composite endpoint of recurrent cerebrovascular events, including TIA, ischemic stroke or intracerebral hemorrhage, and death from cardio- or cerebrovascular cause within 12 months. Cerebrovascular events were defined as those confirmed through hospital work-up guided by clinical and imaging findings, with data retrieved from corresponding discharge summaries. Secondary outcomes included all-cause mortality at 12 months, health-related quality of life using EQ-5D-3L and depression using PHQ-9 with scores ≥ 5 indicating at least mild and ≥ 10 moderate depression [21]. Transition to institutional care as well as functional outcome, defined by a mRS of ≤ 1 for excellent functional outcome and ≤ 2 for functional independence were evaluated at 12 months. Vascular risk factor control was assessed at 3, 6 and 9 months in the SOS-care group and at 12 months in both groups.

### Statistical analysis

Continuous variables were presented as mean ± standard deviation (SD) and non-continuous variables were expressed as median (interquartile range, IQR) or percentages. Between-group differences were assessed using Student *t*-test, Wilcoxon rank-sum test, Chi-square test and Fisher’s exact test. Kaplan–Meier survival curves and Cox regression adjusted for sex and age strata (≤/> 75 years) were used to evaluate the association of SOS-Care with the primary outcome, estimating hazard ratio (HR) and 95% confidence interval (95% CI). Robust Poisson regression was used to evaluate associations of SOS-Care with secondary outcomes, reporting risk ratios (RR) and 95% CI. Cochran's Q Test and McNemar Test assessed differences in vascular risk factor changes across specific time points. Bonferroni-Holm correction was applied for multiple comparisons. Analyses were per-protocol, excluding dropouts and included sensitivity analyses for certain subgroups. Missing data was handled through pair-wise deletion. Statistical significance was set at *p* < 0.05. Analyses used Stata (version 12.1, StataCorp., College Station, TX) and R (version 4.31, R Foundation for Statistical Computing, Vienna, Austria).

## Results

### Study population

**Fig. 1:**
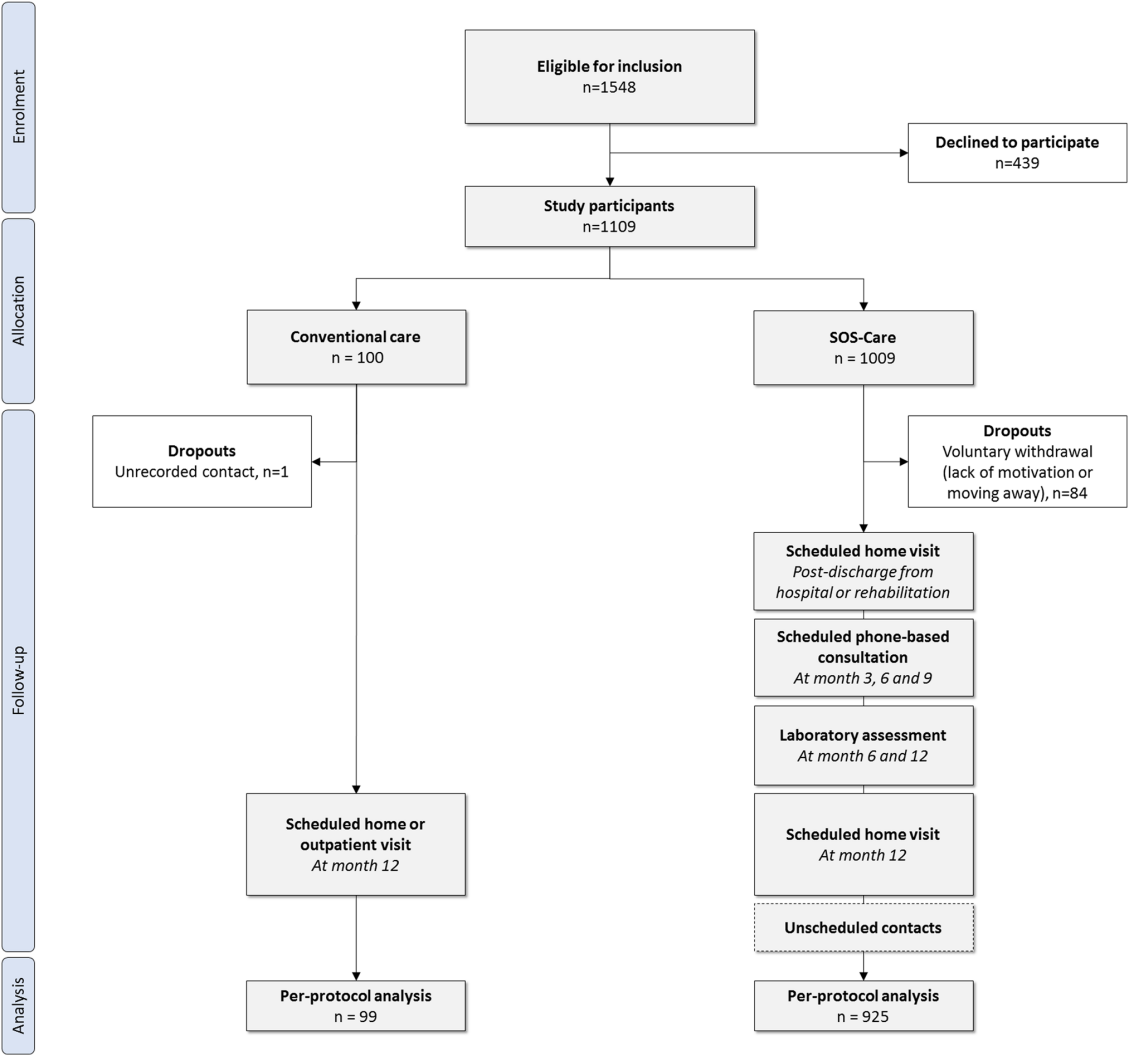
Study flow diagram

Between 11/2011 and 12/2020, 1448 patients were approached for SOS-Care at the two study sites. Of these, 1009 patients consented to participate (Dresden, *n* = 694; Chemnitz, *n* = 315). The mean age was 70.7 ± 12.9 years, 52.7% were male and the median admission NIHSS score was 3 (IQR, 4) points. The most frequent even was an acute ischemic stroke (76.7%) followed by TIA (18.7%) and intracerebral hemorrhage (4.6%). Eighty-four patients (8.3%) withdrew from SOS-Care, primarily due to personal reasons and moving away. The control group was recruited from 10/2016 to 09/2018 and included 100 patients with 85% having an acute ischemic stroke, 10% TIA and 5% intracerebral hemorrhage. One control patient (1%) was lost to follow-up due to unrecorded contact details. Figure 1 illustrates the study flow diagram. A total of 925 patients from SOS-Care and 99 from the control group remained for final data analysis. Baseline characteristics were well balanced, except for a lower proportion of male patients and fewer patients with a history of dyslipidemia in SOS-care compared to controls, as detailed in Table 1.

**Table 1:** Demographics and clinical characteristics of the study population

Variable	SOS-Care *n* = 925	Controls *n* = 99	*p*
Demographics			
Age, years, mean ± SD	70.8 ± 12.7	69.8 ± 11.8	ns
Age ≥ 75 years, *n* (%)	430 (46.5)	39 (39.4)	ns
Male sex, *n* (%)	485 (52.4)	69 (69.7)	0.001^*^
Stroke type, *n* (%)			
Acute ischemic stroke	713 (77.1)	84 (84.9)	ns
Transient ischemic attack	176 (19)	10 (10.1)	ns
Intracerebral hemorrhage	36 (3.9)	5 (5.1)	ns
Admission stroke severity^a^			
NIHSS, median (IQR)	3 (4)	3 (4)	ns
Minor stroke/TIA (NIHSS < 6), *n* (%)	699 (76.1)	78 (78.8)	ns
Reperfusion therapy, *n* (%)^b^			
Intravenous thrombolysis	145 (16.4)	17 (18.1)	ns
Endovascular therapy	58 (9.9)	7 (7.5)	ns
Vascular risk factors, *n* (%)^c^			
Arterial hypertension	764 (84.7)	81 (81.8)	ns
Diabetes mellitus	313 (34.7)	31 (31.3)	ns
Dyslipidemia	520 (57.8)	39 (39.4)	< 0.001^*^
Tobacco use	167 (18.5)	22 (23.9)	ns
Atrial fibrillation	210 (23.3)	24 (24.2)	ns
Depression	65 (8.4)	9 (9.1)	ns
Discharge disposition, *n* (%)^d^			
Rehabilitation	316 (49.1)	39 (39.4)	ns
Institutional care	1 (0.2)	1 (1)	ns
Home	319 (49.5)	45 (45.5)	ns
Inter-hospital transfer	8 (1.3)	14 (14.1)	< 0.001^*^
mRS at discharge, median (IQR)^e^	2 (3)	2 (3)	ns

*NIHSS* National Institutes of Health Stroke Scale, *TIA* transient ischemic attack, *mRS* modified Rankin Scale

^a^Data available for 919 SOS-Care patients

^b^Data on intravenous thrombolysis available for 922 SOS-Care patients and on endovascular therapy for 623 SOS-Care patients, all of whom had ischemic stroke or TIA

^c^Data on hypertension, diabetes and atrial fibrillation available for 902 SOS-Care patients; on dyslipidemia for 900 SOS-Care patients; on tobacco use for 903 SOS-Care patients and 92 controls; and on depression for 771 SOS Care patients

^d^Data available for 644 SOS-Care patients

^e^Data available for 794 SOS-Care patients

^*^Significance determined after applying Holm–Bonferroni correction

### Primary outcome

**Fig. 2:**
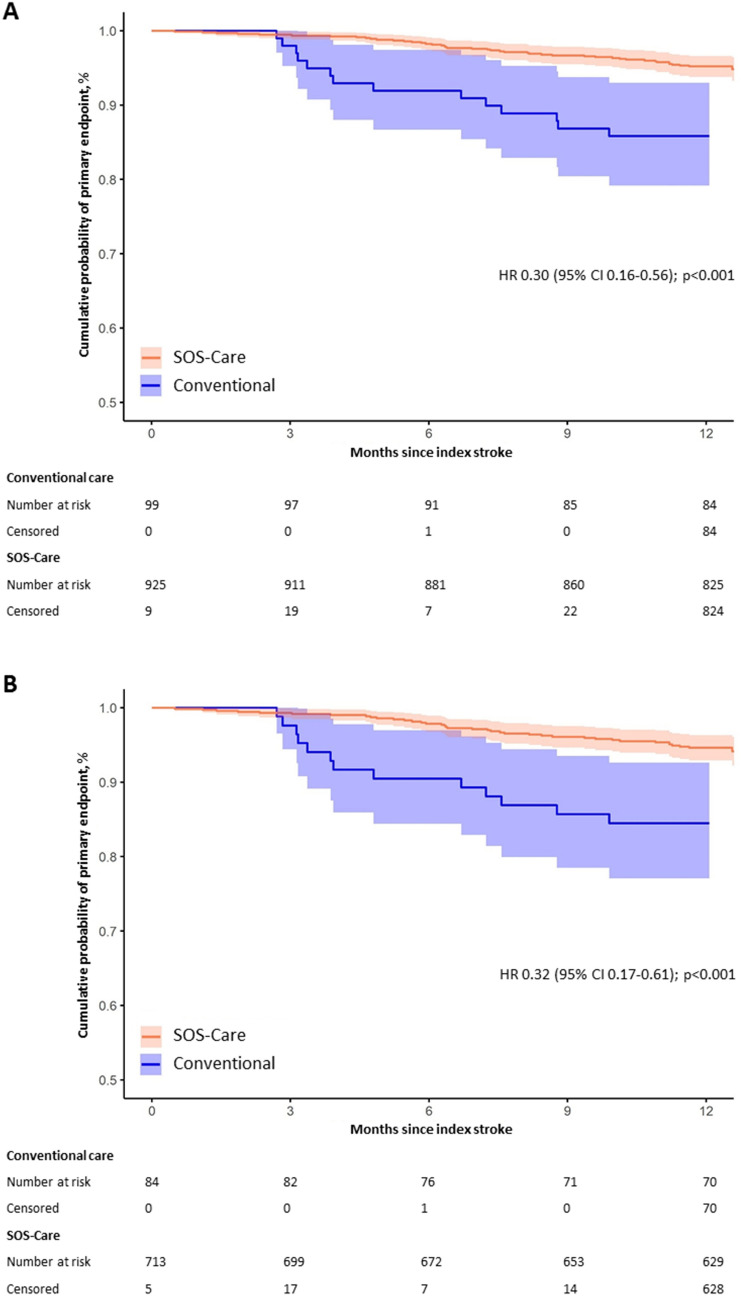
Kaplan–Meier curves for the primary outcome. Kaplan-Meier curves for the cumulative probability of the primary outcome (composite of stroke, TIA or vascular death) for the entire study population (**A**) and for patients whose qualifying event was an ischemic stroke (**B**)

The cumulative risk of recurrent stroke, TIA or vascular death within the 12-month follow-up, was significantly lower in the SOS-Care group compared to the control group (HR 0.30, 95% CI 0.16–0.56, *p* < 0.001). The Kaplan–Meier survival curves are depicted in Fig. 2. Details on these outcomes are presented in Table 2. The sensitivity analysis of the 203 SOS-Care patients recruited at Dresden study site concurrently with the controls showed comparable results (HR 0.28, 95% CI 0.12–0.65, *p* = 0.003). Additional analyses of recruitment periods before (HR 0.27, 95% CI 0.12–0.59, *p* < 0.001) and after (HR 0.08, 95% CI 0.02–0.35, *p* < 0.001) the control group recruitment period also aligned with these findings. In addition, the intervention effect was also evident when considering only patients recruited at Chemnitz study site (HR 0.49, 95% CI 0.24–0.97, *p* = 0.04).

**Table 2:** Primary and secondary outcomes

Variable	SOS-Care *n* = 925	Controls *n* = 99	*p*
Composite endpoint, *n* (%)	45 (4.9)	14 (14.1)	< 0.001^*^
Recurrent stroke/TIA	44 (4.8)	14 (14.1)	< 0.001^*^
Vascular death	3 (0.3)	3 (3)	0.014^*^
All-cause death, *n* (%)	51 (5.5)	4 (4)	ns

*Significance determined after applying Holm–Bonferroni correction

*TIA* transient ischemic attack

When the analysis was restricted to 713 SOS-Care and 84 control patients who had an acute ischemic stroke as their qualifying event, the intervention effect on the primary outcome persisted (HR 0.32, 95% CI 0.17–0.61, *p* < 0.001). This result remained consistent across subsequent sensitivity analyses, which considered only ischemic stroke patients from SOS-Care recruited at Dresden study site concurrently with the control group or during recruitment periods before and after the control group recruitment period. However, the intervention effect did not reach statistical significance (HR 0.49, 95% CI 0.24–1.0, *p* = 0.052) when the analysis was limited to ischemic stroke patients recruited solely at Chemnitz study site.

### Vascular risk factor control

**Fig. 3:**
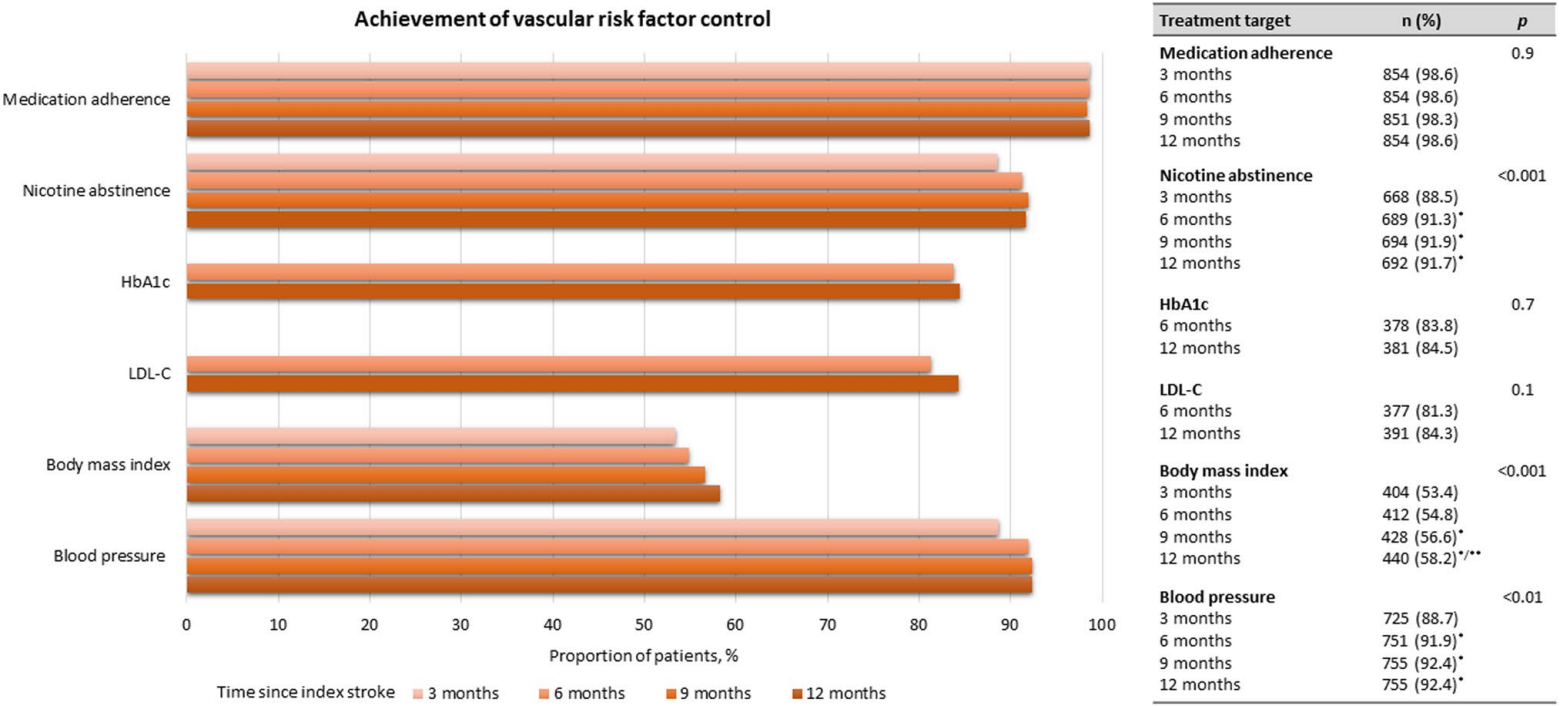
Vascular risk factor control among SOS-Care patients**.** *p < 0.05 after Holm-Bonferroni correction for pair-wise comparisons, using the 3-month time point as reference; **p < 0.05 after Holm-Bonferroni correction for pair-wise comparisons, using the 6-month time point as reference. ^§^based on 866 patients;^†^based on 755 patients; ^‖^based on 451 patients; ^‡^based on 464 patients; ^$^based on 756 patients; ^‡‡^based on 817 patients

At 12 months, patients who completed the post-stroke program demonstrated significantly higher achievement rates in treatment goals for arterial hypertension (91.4 vs. 55.6%; *p* < 0.001), LDL-C (82.3 vs. 69.5%; *p* = 0.017), HbA1c (85.5 vs. 72.3%; *p* = 0.017), BMI (54.8 vs. 33.7%; *p* < 0.001) and adherence to secondary prevention medication (98.6 vs. 75.8%; *p* < 0.001) in comparison to controls. There was no difference in the proportion of nicotine abstinence between the two groups (91 vs. 87.8%; *p* = 0.31). Figure 3 provides an overview of vascular risk factor control across all pre-scheduled visits in the SOS-Care group.

### Secondary outcomes

At 12 months, EQ-5D-3L (available for 527 SOS-Care and 75 controls) and PHQ-9 (available for 685 SOS-Care and 76 controls) scores showed no significant differences between SOS-Care and control patients (0.79 ± 0.22 vs. 0.81 ± 0.21, *p* = 0.3 and 6.06 ± 4.96 vs. 7.04 ± 5.74, *p* = 0.1, respectively). An evaluation across the five dimensions of the EQ-5D-3L did not reveal positive effects either. Furthermore, the proportion of patients with at least mild or moderate depressive symptoms were also comparable between both groups (55.6 vs. 60.5%; *p* = 0.41 and 20.7 vs. 27.6%; *p* = 0.16, respectively).

While there was no significant difference in excellent functional outcome at 12 months (63.7 vs. 60%; *p* = 0.48), more SOS-Care patients achieved functional independence (82.2 vs. 72.5%; *p* = 0.03). This difference remained significant after adjusting for age, baseline NIHSS and stroke type (RR 1.14, 95% CI 1.01–1.29; *p* = 0.03). No difference was found in institutional care at 12 months between SOS-Care and control patients (8.9 vs. 12.4%; *p* = 0.26), although a trend suggested a decreased risk with the intervention after adjusting for age, baseline NIHSS score and stroke type (RR 0.60, 95% CI 0.34–1.06; *p* = 0.079).

### Case manager interactions

The stroke-case managers maintained 24.4 ± 12.9 interactions per patient, including 4.0 ± 1.8 in-person and 20.4 ± 12.2 through phone or email. Each patient required an average of 2.6 ± 1.7 interventions involving secondary prevention medication adjustments, coordination with primary care physicians (e.g., hospital admissions) and guidance for social needs.

## Discussion

This prospective cohort study suggests that case management-based post-stroke care may lower the risk of recurrent stroke and vascular death in patients with TIA, ischemic and hemorrhagic stroke. This association could be attributed to effective management of vascular risk factors like hypertension, dyslipidemia, diabetes and obesity, and improved adherence to secondary prevention medication. Although the program did not reduce overall mortality, its particular impact on vascular outcomes underscores its potential role in addressing mortality risks after stroke.

Most recent post-stroke care programs predominantly relied on outpatient care [14–16]. The randomized INSPiRE-TMS trial investigated an organized support program that included eight outpatient visits over a two-year period following TIA and non-disabling minor stroke [14]. Similarly, the SANO trial provided five outpatient visits over 12 months for ischemic stroke patients, primarily focusing on organizational and behavioral aspects [16]. The STROKE-CARD trial offered a single outpatient visit three months after TIA or ischemic stroke in addition to a web-based patient portal for stroke education and risk factor monitoring [15]. Although these programs provided multiprofessional stroke expertise through in-person consultations, their accessibility may be limited for elderly patients or those with substantial disabilities, who often have mobility challenges. Notably, most trials excluded patients from their post-stroke care program whose disabilities could hinder access to outpatient care [14, 15]. Conversely, younger patients and those with TIA or minor stroke may have easier access to ambulatory stroke services or be more effective at implementing secondary prevention strategies provided by their primary care physicians, as observed in the INSPiRE-TMS trial, where primary care physicians played a key role in treating control patients with TIA or minor stroke [14].

Our post-stroke care program adopted an inclusive approach, enabling patients to participate regardless of their mobility or residence status. The needs of patients with substantial disabilities or those necessitating constant care including nursing home residents may differ significantly from those with non-disabling strokes. In addition, our program encouraged patients to actively engage with their assigned stroke-case managers. This interaction was not limited to pre-scheduled home and phone visits, but also included addressing medical or social needs as they arose. The high frequency of 24 case manager interactions per patient, reflects the extensive and diverse needs in post-stroke care and likely contributed to sustainable problem resolution in our study patients [22]. Although our cohort eventually comprised patients with lower stroke severity (76% of SOS-Care patients and 79% of control patients had an NIHSS < 6 points), the program was not restricted to patients based on stroke severity, stroke type or care requirements. Inclusivity appears beneficial in post-stroke care as younger patients with milder deficits may more frequently require psychologic support and assistance in social reintegration, whereas patients with severe deficits rather require focused management of stroke-related complications like spasticity.

The 4.9% rate of recurrent stroke or vascular death at 12 months in our SOS-Care post-stroke population was lower than rates reported in the general stroke population and comparable to those observed in the STROKE-CARD (5.4%) and SANO (5.3%) trials [15, 16, 23]. These comparisons suggest that interventions in the latter trials may have had some effect, despite the lack of differences when compared to similarly low event rates in the control groups. Moreover, while recent post-stroke care trials have shown effectiveness either in controlling risk factors or in reducing vascular events, the absence of a correlation between these outcomes warrants further exploration. One possible explanation could be the infrequent assessment of risk factors in control groups across most trials. Commonly, assessments were only conducted at baseline and at 12 months, providing a limited snapshot of the overall risk factor status. This could potentially obscure any interim changes in the control group, thereby limiting the interpretation of corresponding vascular risks. In our study, although risk factor assessment for controls was also limited to the 12-month visit, the intervention group showed effective management of key risk vascular factors such as HbA1c, LDL-C and blood pressure throughout the program, with more than 80% of participants achieving target goals at each visit. In contrast, the INSPiRE-TMS and SANO trials reported somewhat less control over certain risk factors at the 12-month follow-up [14, 16].

Given that over 30% of stroke survivors experience post-stroke depression in the first year, it is essential for post-stroke care to address emotional and psychosocial long-term sequelae of stroke [24, 25]. In our study, at least one in five patients had PHQ-9 scores suggestive of depression in the first year post-stroke, a rate higher than the 4% reported in the SANO trial [16]. This emphasizes the need for effective strategies to evaluate and manage mental health post-stroke, considering its significant impact on patient outcomes and recurrence risk [25, 26]. Our study found no significant differences in health-related quality of life between program participants and controls, consistent with results from the INSPiRE-TMS trial [14]. However, the STROKE-CARD trial observed 43% of its participants achieving the highest EQ-5D-3L scores vs. 32% in the control group [14, 15]. This difference might be linked to the use of a web-based patient portal for monitoring post-stroke complications and addressing social needs, which is associated with improved quality of life in stroke survivors [22]. While the supplementary use of digital health applications in post-stroke care might pose challenges for elderly or nursing care-dependent patients, they harbor great potential in aiding the recovery of stroke patients. Currently, a digital application utilizing sensor-based technology and mobile devices to monitor physical activity, blood pressure, and electrocardiographic signals, combined with personalized case management-based post-stroke care, is undergoing evaluation in a feasibility trial [12].

Our study has limitations. First, its non-randomized design may have affected internal and external validity. There is potential for selection bias as patients with higher health awareness might have been more inclined to participate in the post-stroke program and factors such as ethnicity, level of education and language spoken may have further influenced the outcomes of our study—common issues in voluntary health programs. We did not collect specific data on these variables, which limits our ability to assess their impact on the study outcomes. In addition, two-thirds of post-stroke care participants were insured by a specific health insurance provider. Although this provider represents nearly 50% of the study region’s population, this factor may still have influenced the generalizability of our findings [17]. Regional variations in healthcare stroke systems may have further limited generalizability. Third, changes in guideline-based target goals for vascular risk factors and evolving stroke-reperfusion therapies during the 10-year study period may have affected stroke outcomes especially in the intervention group. Nonetheless, the sensitivity analysis focusing on the narrower controls recruitment period confirmed our overall findings. Fourth, lack of data on behavioral and cognitive recovery aspects limits our ability to assess the program’s effectiveness in this context. However, acknowledging this limitation, we have recently extended our ongoing program to include assessments of frequent but commonly unrecognized post-stroke complications like fall-related anxiety and spasticity [22]. Fifth, the control group was notably smaller compared to the intervention group. This imbalance occurred because the program was not funded as a research project, limiting personnel resources for control patient observations. However, baseline characteristics were well balanced between SOS-Care and control patients. In addition, there was a relatively high proportion of missing data concerning secondary outcomes, leaving uncertainties in these results, yet this appears to be a common challenge in post-stroke care [14–16]. Lastly, the program’s duration of 12 months limits our understanding of the long-term effects of our post-stroke care model.

The strengths of this study include the large cohort of stroke patients supported through personalized case management, the provision of real-world data on the long-term applicability of post-stroke care—making it the longest study period in this field to date and the first program partially funded by health insurance and thereby integrated into regular healthcare.

## Conclusions

Case management-based care might be effective in mitigating the risks of stroke recurrence and vascular death post-stroke. Future trials should also focus on quality-adjusted life years and cost-effectiveness analyses to further explore the patient-centered benefits of case management-based post-stroke care models.

## Data Availability

The datasets used and analyzed during the current study are available from the corresponding author on reasonable request.
